# Anterior Shoulder Dislocation during Breaststroke Swimming Technique: A Case Report and Review of the Literature

**DOI:** 10.1155/2019/9320569

**Published:** 2019-04-09

**Authors:** Angelo V. Vasiliadis, Christos Kalitsis, George Biniaris, Antonios Saridis

**Affiliations:** ^1^Department of Orthopaedic Surgery, General Hospital of Katerini, Katerini, Greece; ^2^Aristotle University of Thessaloniki, Thessaloniki, Greece

## Abstract

A 36-year-old woman presented with anterior shoulder dislocation as a result of breaststroke swimming training. She complained of pain and restriction of movement. A radiograph revealed the shoulder was dislocated, and the patient was treated successfully with closed reduction. The mechanism of injury seemed to be a relation between the initial pull phase of breaststroke technique and the weakest position of the shoulder in extension and external rotation. In our experience, if a patient has a history including a shoulder dislocation, this needs to be considered carefully before incorporating aquatic therapy into the rehabilitation program. Attention must also be given to the crucial timing of initiating swimming training.

## 1. Introduction

Shoulder dislocation is the most common major joint dislocation encountered in the emergency departments, with over 85% of traumatic glenohumeral dislocations being anterior [1]. A recurrent dislocation is the main complication after anterior dislocation of the shoulder, with redislocation rates ranging from 4% to 96%, influenced by a variety of factors, such as age, gender, sports participation, and associated lesions [2]. Both contact sports, such as football and wrestling, and noncontact sports like tennis, swimming, and volleyball are predisposed to this type of injury [3, 4]. Anterior shoulder dislocation (ASD) most commonly occurs either as a result of a sudden trauma or from an underlying shoulder joint instability in the young male population and usually results in specific structural damage to the bone, labrum, and joint capsule, such as Bankart lesions and the Hills-Sachs lesion [1].

A closed manipulative reduction under pain relief in the emergency department is the initial treatment of choice. The ensuing treatment of ASD is complex and challenging and should be tailored to each individual's age, occupation, and degree of physical activity [2]. Conservative management usually requires a rest period with arm immobilization in a sling, followed by a well-supervised rehabilitation program, while surgical treatment should be recommended in active patients to reduce the risk of recurrence [1]. During the rehabilitation period, physical and aquatic therapy focus on regaining motion, shoulder strength, scapular stabilization, and preventing a repeated shoulder dislocation. The advisability of the addition of aquatic therapy or swimming to the rehabilitation recommendations following reduced shoulder dislocation seems to be unclear. Thus, the purpose of this study is to present a classic case of an anterior shoulder dislocation, describe the nature and the mechanism of the injury, and review the literature concerning the initiation or not of the aquatic therapy/swimming in the early stage of rehabilitation treatment.

## 2. Case Report

**Figure 1:**
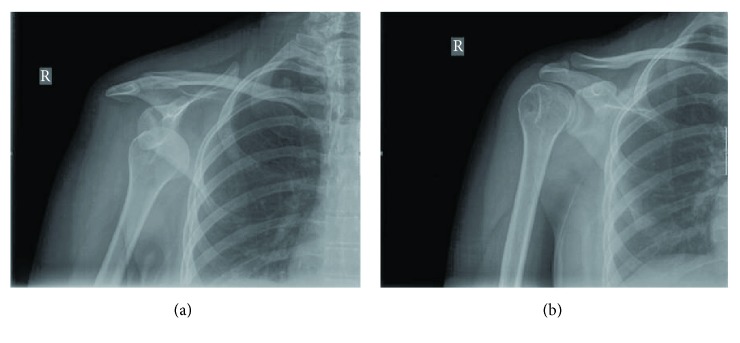
Radiographs of the shoulder demonstrate anterior shoulder dislocation (a) and her postreduction (b).

A 36-year-old right-handed female tourist was admitted to the emergency department with her arm held in external rotation, complaining of severe pain and inability to move her right shoulder, which occurred while swimming breaststroke technique in the sea. After 15 minutes of breaststroke swimming, she suddenly felt her shoulder going out of place and was unable to continue swimming. In the initial physical examination, the shoulder joint was in slight abduction and external rotation. Her right shoulder had a typical “squared-off” appearance, with a prominence of the acromion. A careful neurovascular assessment proved normal. A radiograph of her right shoulder showed anterior dislocation (Figure 1(a)). The patient was sedated with pethidine (100 mg in 2 ml), and reduction was attempted. The right shoulder was easily reduced using Kocher's technique and confirmed by radiograph (Figure 1(b)), and her arm was immobilized (in adduction and internal rotation) in an arm sling. Our written discharge instructions pointed out the need for the restriction of arm movement, a magnetic resonance imaging in order to evaluate the soft-tissue structures, and an orthopaedic follow-up one week later in her home country.

The patient's history revealed a longstanding antiepileptic treatment period, recreational swimming participation, and one previous incidence of right ASD 3 months previously. The patient had received regular physiotherapy in her home country, and she had followed a scheduled rehabilitation program with swimming breaststroke technique (from the 10th week of her rehabilitation program and after) in order to follow this program during her 10-day vacation in Greece.

## 3. Discussion

ASD is the most frequent joint dislocation treated in the emergency department, with the reported recurrence rate varying widely and influenced by many different risk factors, such as young age, participation in high-stress sports, and a previous history of structural joint damage [1]. Rhee et al. [2] reported an average interval between the first and second shoulder dislocation of 29 weeks, with 33.7% of them experiencing a redislocation from 3 months to one year later. In the same study, athletes seemed to have a shorter interval (13 weeks) between the first and second dislocations compared to nonathletes (approximately 34 weeks). Our study revealed a second shoulder dislocation in a young female recreational swimmer which occurred while she was performing breaststroke swimming technique as part of her rehabilitation treatment protocol 13 weeks after her first episode.

**Figure 2:**
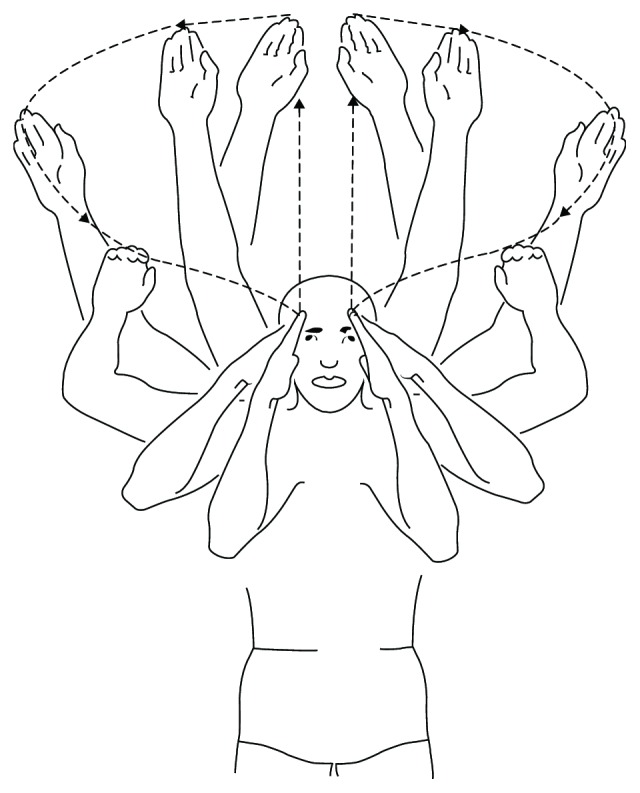
Shoulder girdle motion during the pull phase of a breaststroke swimming stroke. The dotted lines with black arrows show the direction of hands while performing breaststroke swimming.

Swimming, as an overhead sport activity, requires repetitive high load shoulder movements involving continuous humeral circumduction, and consequentially, a shoulder dislocation tends to be a common injury [5]. Dlimi et al. [6] described a bilateral ASD at the start of a backstroke competition, in which the swimmer pushed his hands away from the block, swung his arms around sideways to the front, and threw his head to the back. Gökkuş et al. [4] reported a 38-year-old male with an inferior shoulder dislocation while swimming in a pool. In our case, the patient, a young woman, dislocated her right shoulder as a result of a breaststroke swimming. She described the feeling, and the sound, of a “pop” during the pull phase of the stroke. In this phase, the arms are moved simultaneously through a motion where the arms begin fully extended out in front, hands pitched outwards and downwards to an angle of about 45 degrees, and then the arms pull outwards and downwards, with the elbows beginning to bend and the shoulders rolling inwards (Figure 2) [5].

Breaststroke swimming requires several different shoulder motions. Starting from a shoulder-flexed posture with elbows extended straight ahead. Then, the shoulders are moved through internal rotation, adduction, and finally extension [7]. During breaststroke swimming, all movements of the arms are simultaneous and on the same horizontal plane without alternating movement. A kinematic analysis of the arm movement during breaststroke swimming shows that in the propulsion phase, the arms start moving outside with the hands twisted out in pronation, continue through the catch and backward arm pull, with the hands then moving inwards until the beginning of the forward hand movement [8].

The mechanism of ASD is usually a sharp stress to an abducted, externally rotated and extended extremity, when the shoulder is in its weakest position [3]. It seems that the initial pull phase in breaststroke swimming, when the athlete obtains their most forward propulsive power, correlates with the weakest position of the shoulder. The force can be strong enough, and due to the combination with its inherent point of weakness, the humeral head is more prone to dislocate. Especially when a shoulder has been previously dislocated, as described in our patient, it is more vulnerable to recurrent dislocation.

Despite the fact that breaststroke swimming is not such an overhead activity compared with other swimming strokes, the possibility of a shoulder dislocation is a reality. The crucial point in our case report is the introduction of breaststroke swimming (in the 10^th^ week) as part of her rehabilitation program after her first episode of ASD. The present data give us a partial and inadequate overview on this subject, and the literature is still not clear on the following aspects: (i) the appropriate time point to start aquatic therapy, (ii) the correct time to begin swimming training, and (iii) the daily frequency of aquatic therapy and swimming training protocols.

**Figure 3:**
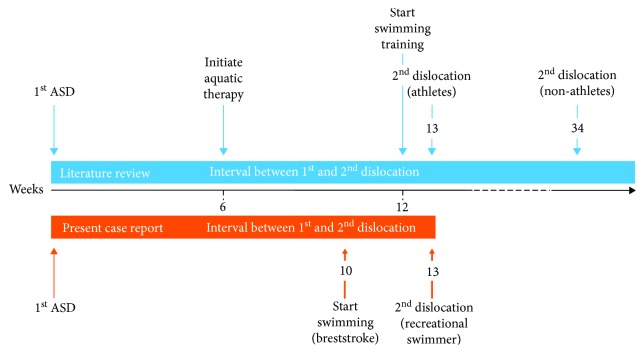
Time points of initiating aquatic therapy and swimming training after anterior shoulder dislocation (ASD) correlated with the lack of a second dislocation, according to an athletic or nonathletic profile. The literature review and our case report are indicated with blue and orange, respectively.

A recent case study has reported that aquatic therapy (twice a week) was initiated after 6 weeks, as part of a rehabilitation program (Figure 3) [9]. Dlimi et al. [6] report that a 20-year-old competitive swimmer, who experienced bilateral anterior dislocation of the shoulders and was treated with closed reduction, was able to resume swimming 3 months later (Figure 3). A recent review article demonstrates that swimming can be initiated earlier, in the second phase of a rehabilitation program (6th week to 3rd month), where the main goal is to regain full shoulder flexion and internal range of motion and be at 90% of full external rotation. A key limitation for starting swimming is that the full overhead strokes may be limited because of decreased strength and scapula stabilization [10]. A careful reintegration back into swimming training is essential. Khodaee et al. [11] indicate a gradual increase in training load. In this study, the authors encouraged a return to the pool, and swimming 1000 – 2000 meters slowly and comfortably, only when the swimmer is able to reach above shoulder height without pain. An addition of 500 meters every 3 workouts is encouraged when the swimmer experiences no pain during resisted motions in all planes. All four competitive swimming strokes and short sprint sets can be integrated once swimming 4000 – 5000 meters is pain free [11].

Movement on the shoulder during breaststroke can vary, with more motion occurring below the surface of the water. Also, breaststroke swimming activates several muscle groups, such as the biceps brachii, triceps brachii, subscapularis, latissimus dorsi, pectoralis major, supraspinatus, infraspinatus, serratus anterior, and deltoid [12]. It is obvious that after a postreduction period of 2-3 weeks, where shoulder movements were restricted, a well-rounded program of strength training is always valuable in order to prevent a recurrent shoulder dislocation. These training programs should focus on external rotation compensatory strengthening, as well as on all joint dynamic and static muscle group stabilizers [13]. It is also indicated that muscular balance of the shoulder complex is of great importance to prevent this type of injury. The proposed preventive activities for coaches include: (i) resistance strength training and balance exercises, (ii) improvement of proprioception and neuromuscular control, and (iii) correction of swimming technique [14].

In the current literature, the experts' opinion seems to be that most athletes are able to return to swimming protocols after 6 weeks, when range of motion and strength have returned to near normal, but there is a high risk of a recurrent shoulder dislocation. Spigelman et al. [15], in a clinical commentary on a return to swimming protocol for competitive swimmers, present two criteria: (i) the swimmer is nearly pain free in the shoulder complex and (ii) full active extension and external rotation of the glenohumeral joint. In the same study, a gradual increase in training workload is indicated in order to prevent overuse injuries. Finally, Pollard and Fernadez [16] conclude in their review that it is important to bear in mind the effects of detraining on the swimmers if they are kept “out of the water” for any substantial period of time. Every effort should be focused on minimizing this period and keeping the competitive swimmer “in the water.”

## 4. Conclusion

Anterior shoulder dislocation is a common condition encountered in the emergency departments by health care physicians. Swimming requires several different shoulder motions with varying degrees of internal and external rotation. Breaststroke swimming is the least overhead stroke compared with other swimming strokes, although the initial pull phase in breaststroke swimming is coincident with the weakest position of the shoulder. Particular attention must be paid when there is a history of an earlier shoulder dislocation and consideration given to the timing of the initiation of aquatic therapy/swimming in a rehabilitation program. An initial protection period is crucial, especially in the first 3 weeks, to allow healing of the damaged soft tissues. The key point to prevent a recurrent dislocation is strengthening exercises to be comfortable and progress gradually. In order to return to swimming protocols, recreational swimmers and athletes should be pain free during the activities of daily living and have a normalized range of motion, while strength of injured side must be almost equal to the uninjured contralateral side.
